# Optimizing Sanitation Network Upgrading Projects in Slum Areas

**DOI:** 10.1007/s11524-023-00751-w

**Published:** 2023-08-03

**Authors:** Mohamed Marzouk, Mahmoud Bahi, Omar El-Anwar

**Affiliations:** Structural Engineering Department, Faculty of Engineering, Cairo University, Giza, Egypt

**Keywords:** Slum area, Sanitary network upgrading, Network logistic analysis, Serviceability performance measurement, Genetic algorithms

## Abstract

Infrastructure upgrading projects are a key element in enhancing the livelihood of residents in slum areas. These projects face significant constructability challenges common to dense-urban construction coupled with the unique socioeconomic challenges of operating in slums. This research focuses on sanitation network upgrading projects in slum areas and proposes a novel methodology capable of (1) accounting for the unique constructability challenges for these projects, (2) accelerating the provision of sanitation services, and (3) optimizing construction decisions. The key contribution of this research to the body of knowledge is in developing a comprehensive construction planning framework capable of achieving these three objectives. The proposed framework focuses specifically on sewer lines upgrading within the larger sanitation networks upgrading projects. This framework consists of five main models that can guide planners in selecting the appropriate equipment sizes, trench system configuration, and optimal equipment routing, in addition to identifying all possible execution sequences along with the corresponding construction cost and duration of each sequence. Most notably, this framework proposes an approach to assess the serviceability of different construction plans measured by how fast sanitary services can be provided to slum dwellers. A multi-objective, genetic algorithms optimization model is developed to identify the optimal construction plans that accelerate the sanitary service provision to residents while minimizing construction costs. A real-world example is presented to demonstrate the model capabilities in optimizing construction plans.

## Introduction

There is a continuous growth in urban population around the world. The high concentration of residents in densely populated areas increases the demand for development projects to serve the growing social and economic needs. Hence, many infrastructure and public construction projects take place in congested urban sites, which in turn impose new construction management requirements for urban construction [1, 2]. From a developmental perspective, one of the most challenging types of densely populated areas are slums, whose unplanned nature adds another level of complexity to the construction process [3]. Around 24% of the world’s urban population lives in slums, with the number of slum dwellers estimated at one billion people [4]. This number is expected to grow in the decades to come [5]. Living in such unplanned urban zones poses a number of challenges that require immediate intervention. Among these challenges are as follows [6]: (1) the poor, or even unsafe, housing conditions; (2) the lack of basic social services; (3) the lack of clean water and sanitary facilities; (4) overcrowding; and (5) high levels of poverty; among other issues.

Optimizing sanitation network design has been receiving significant attention in research studies. For example, Afshar [7] developed a genetic algorithm to optimize the nodal elevations of storm water networks. Pan and Kao [8] utilized genetic algorithms and quadratic programming to optimize pipe diameters, slopes, and depths in a sewer system as well as the system cost. Similarly, Haghighi and Bakhshipour [9] developed an adaptive genetic algorithm to optimize the sewer pipes diameters and slopes and the pump indicators. Moeini and Afshar [10] presented an adaptation of the ant colony optimization algorithm with a tree growing algorithm to optimize the layout and pipe sizes. Furthermore, Safavi and Geranmehr [11] proposed a mixed-integer linear programming model to minimize the costs of pipe purchase, pipe-laying, and manhole construction, while meeting the allowable pipes slopes, velocities, and relative depths for the sewage discharge rates. Mixed-integer linear programming has also been used to optimize sewer layouts in order to minimize the total cost required for constructing pipes [12]. Zaheri et al. [13] proposed a cellular automata based simulation-optimization approach to optimize the design of household sewer networks. The approach includes optimizing the diameters and nodal elevations of each pipe. More recently, Duque et al. [14] proposed a mathematical optimization framework to optimize the sewer network layout selection and hydraulic design in order to optimize costs. It uses mixed-integer programming to determine the flow rate and direction per pipe and uses a shortest path algorithm to the diameter and the upstream and downstream invert elevations of pipes.

Other research studies focused on optimizing decisions related to the asset management of sanitation networks. For example, Abraham et al. [15] discussed the major aspects of integrated management for sewer systems. This integrated management approach included using optimization to maximize the benefit/cost ratios over a planning horizon. It also discussed network identification, sewer classification and condition rating systems, and sewer mechanisms and prediction modeling. Later, Halfawy et al. [16] presented a multi-objective genetic algorithm optimization technique to select the set of sewers to be renewed each year. This approach takes into account the associated costs, condition improvement, and risk reduction. From a different perspective, Ermolin [17] proposed a mathematical model to minimize the electric energy consumption by the sewer network pumping stations.

The aforementioned studies offered significant contributions to the design and asset management of sewer networks. Similar efforts are needed to optimize the construction plans needed when constructing these projects. However, capturing the knowledge necessary to optimize construction means and methods is a challenging task, both for sewer networks and other construction works. This knowledge is typically maintained in the form of personal experience with a lack of formal systems to collect and manage this kind of knowledge [18].

Roy and Lees [19] developed an agent-based model to model current existing social, economic, and environmental situation impacts in slums based on 37 slums in Bangalore. They modeled stabilizing effect of social satisfaction and concluded that it decreases as a function of the number of social contacts that move. Optimizing construction planning becomes even more challenging in slums. In slum areas, infrastructure upgrading projects experience significant constructability challenges, which can be attributed to two main sources [20, 21]. The first source of challenges is the dwellers’ resistance to the upgrading projects, which in some cases can pose serious safety risks to the construction personnel. This resistance is attributed to the fact that the execution phase of the construction projects can disrupt the livelihood of surrounding residents, and in some cases even require temporary relocation of residents. The second source of challenges is logistical in nature. These challenges stem from the over-crowdedness and unplanned nature of the slum area, and they are typically manifested in the limited accessibility of crews and equipment to the construction sites, lack of storage areas for construction materials, and the need to use inefficient and manual construction methods. The constructability challenges to slum upgrading projects and their consequential risks to the projects’ time and cost have been discussed in previous studies [3, 22]. Accordingly, the upgrading of informal urban areas should be considered a sociotechnical process to realize a comprehensive perspective of its ongoing execution dynamics [23]. While relevant research contributions aimed at identifying the sources of constructability challenges associated with infrastructure upgrading projects in slum areas, there are limited attempts to overcome the aforementioned constructability challenges, especially in sanitation network upgrading projects [23, 24].

It is noteworthy that some research studies address the sanitation problem in slums by discussing non-traditional sanitation alternatives. For instance, Katukiza, Ronteltap [25] proposed a technology selection method for slums. These technologies include Urine Diversion Dry Toilets and biogas latrines. Alternative sustainable sanitation technologies have also been discussed by Katukiza et al. [26] and included anaerobic co-digestion for treatment of excreta and organic solid waste for energy recovery as an alternative to composting in addition to soil and sand filters for grey water. On the other hand, Schouten and Mathenge [27] investigated the appropriateness of communal sanitation facilities in slums. While offering interesting alternative solutions, traditional sanitary upgrading projects still play a key role in slums intervention strategies worldwide. Despite the significance of the aforementioned studies in this section, they do not consider the constructability challenges associated with sanitary upgrading projects in slums and other unplanned areas. There is a need for a construction planning approach capable of considering the human and logistical challenges associated with sanitary upgrading projects in slum areas.

Previous studies provided valuable contributions in the following areas: (1) describing the condition of slums infrastructure and the slum dwellers’ need for basic services; (2) emphasizing the importance of sanitation upgrading projects to slum dwellers; and (3) investigating the unique constructability challenges facing slums upgrading projects. Despite these significant contributions, there is no reported research that investigates how to account for the constructability challenges associated with sanitary upgrading projects in slum areas, including both logistical challenges and dwellers’ resistance. Most existing studies are dedicated to investigating the social, economic, health, and design aspects of slums, with less focus on the construction phase of slums upgrading. These aspects are of great importance; however, the success of the construction phase of the upgrading initiative is as essential, because it is the construction phase that eventually shapes the final outcome of the infrastructure upgrading project [3]. The complexity of the slum conditions has required dedicated research to investigate the social, economic, health, and design aspects of slums; and it is the same complexity that requires dedicated research for the construction phases of slum upgrading projects. While infrastructure upgrading projects in slum areas share some common challenges, there are unique challenges facing each type of infrastructure upgrading project. Given the focus of this paper, a detailed discussion of the unique challenges facing sanitary upgrading projects (compared to other infrastructure upgrading projects) is presented later in the Research Framework section. Accordingly, there is a need for a comprehensive construction planning framework designed specifically to address the unique challenges facing sanitary upgrading projects in slums.

The objectives of this research are threefold: (1) to account for the unique constructability challenges when planning for sanitation upgrading projects in slum areas; (2) to accelerate the provision of sanitation services to slum dwellers; and (3) to develop a model capable of optimizing construction decisions considering constructability challenges and service provision. The key contribution of this research to the body of knowledge is in developing a comprehensive construction planning framework capable of achieving these three objectives. This framework focuses specifically on sewer lines upgrading within the larger context of sanitation networks upgrading. This research represents the first reported attempt to develop such a framework and operationalize it using an optimization model. It is expected that future research studies will build on this framework and adopt similar methodologies to address the constructability challenges facing other types of slums upgrading projects.

The following sections introduce the theoretical framework to achieve these objectives, present the proposed optimization model, and demonstrate the implementation of the proposed framework and associated model using a real-world example. Another key contribution of this research is in presenting an objective metric capable of assessing the rate of sanitary service provision to slum dwellers. This metric is the serviceability index as presented in detail in later sections. The implementation of this metric was necessitated by the need to identify construction plans that would minimize the period of socioeconomic disruptions to dwellers and hence mitigate their possible resistance to the upgrading project.

## Research Framework

A research framework is developed to achieve the aforementioned objectives by addressing a number of research and tactical questions essential for the sanitation upgrading projects in slum areas. First, to enable the construction planning process to account for the unique constructability challenges in sanitary upgrading projects, the following questions need to be addressed: (1) given the accessibility limitations within the slum area, how to identify which pieces of construction equipment are compatible with the existing road network characteristics? (2) Which trench excavation system is most suitable and most economic for each road segment? (3) For each set of possible construction alternatives, how to quantify the cost and duration associated with sanitation upgrading work? (4) How to identify the possible construction sequences within the network? And which sequences are mandatory given the network characteristics and which sequences are recommended? (5) And how to assess the serviceability performance of each proposed construction plan in terms of speed of sanitation services provision? And would there be significant cost tradeoffs among these plans?

**Fig. 1:**
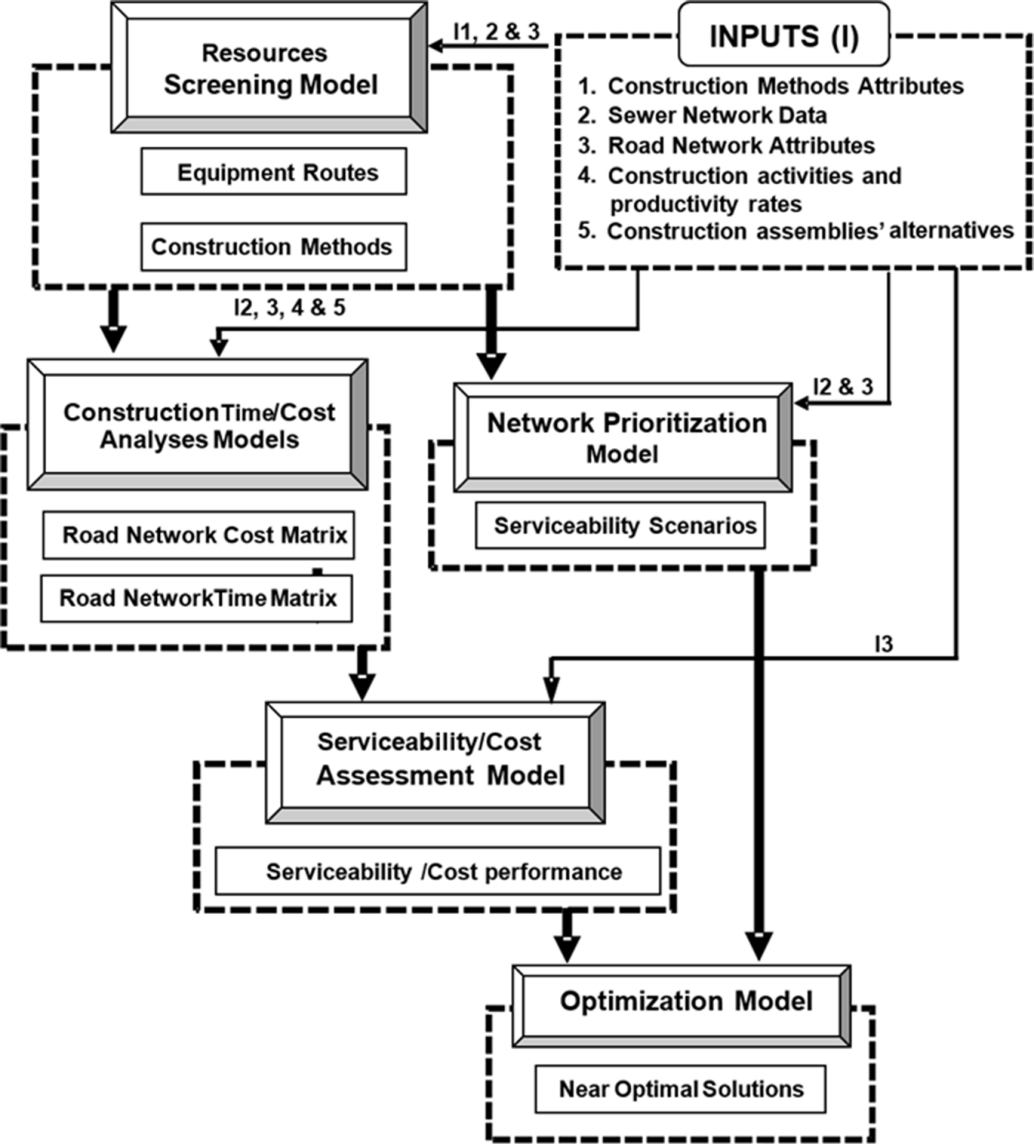
Schematic diagram of framework components

To address the abovementioned five questions, five models are developed within the proposed framework corresponding to each question. As shown in Fig. 1, these models are the resource screening model, trench system configuration model, construction time and cost analyses model, network prioritization model, and the serviceability and cost assessment model. These models are explained in detail in the following subsections and consider five key construction and slum characteristics: namely, construction methods, road network logistics, road network relationships, network design requirements, and demographics.

Before explaining the details of each model, there are two important discussions presented in this section. First, the unique characteristics of sanitary upgrading projects are presented as well as their unique constructability challenges and opportunities. Second, the reasons for residents’ resistance to upgrading projects are discussed. These two discussions are presented to explain how the implementation models address them before explaining the details of each model because these two topics span over all models and are not addressed exclusively by any one model.

### Characteristics of Sanitary Upgrading Projects

Sanitary upgrading projects are a type of infrastructure upgrading, and hence, they face challenges common to infrastructure projects in slum areas. However, there are unique characteristics that distinguish sanitary upgrading projects, some of which offer additional challenges while others offer unique opportunities that can be leveraged for sanitary upgrading projects. These unique characteristics are incorporated into the proposed framework and implementation models, as discussed in the following points.

First, in sanitary upgrading projects, the key source of disruption to the livelihood of residents is attributed to the earthwork and road closures associated with the upgrading works. The negative effect of this issue is exacerbated by the fact that in some cases the impacted roads are in acceptable condition and that the upgrading project is addressing a sanitation problem and not necessarily a road condition problem. Hence, a road closure is not something that all residents are willing to accept, especially if they are not the beneficiary of the sanitation services provided (such as residents who use these roads but do not live in the upgraded area). As such, to minimize the possible resistance to the upgrading project, the construction plans should (1) minimize the duration and scope of road closures to lessen the impact on residents who depend on these road segments in their commute and (2) accelerate service provision to some residents to showcase the value that the upgrading project brings, and hence gain the acceptance of residents who live in the impacted area. The proposed five models address these issues as explained later in their respective sections.

Second, sanitary upgrading projects have an advantage in the fact that service could be turned on for downstream segments of the network if they are connected to the outlet. This allows some residents to start benefiting from the upgrading work in early project phases without waiting for the project to be completed. Subsequently, service can be turned on to their immediate upstream segments and so on. This is an important characteristic of sanitary network upgrades which can be leveraged to gain residents’ satisfaction and lessen their resistance. The proposed framework leverages this characteristic as explained in detail in the “Network Prioritization Model” and “Serviceability and Cost Assessment Model” sections.

Third, a unique characteristic in sanitary networks is that flow is governed by gravity (compared to water networks where flow is typically pressurized or to roadway construction where uphill grades are allowed up to specific slope angles). This characteristic poses an important constraint that needs to be considered when sequencing work. As such, the flow direction is a key element in defining which road segments should be upgraded first, as is explained in detail in the “Network Prioritization Model” section. Fourth, the scope of sanitary upgrading projects not only includes work specific to the sanitation network (such as pipelaying activities, manhole installation, etc.), but also earthwork and road rehabilitation work. Accounting for the complete scope of activities is important to compute a realistic duration and cost for each road segment. Furthermore, there are various construction means and methods that could be adopted for these activities. These considerations are incorporated in the proposed models, as described in the “Trench System Configuration Model” and the “Construction Time and Cost Analysis Model” sections.

Fifth, and to build on the previous points, sanitary projects—as well as other infrastructure upgrading projects—experience challenges in slum areas, such as very narrow roads, possibly unstable structures surrounding the road where excavation work needs to take place, and limited access points to the road network, to name a few. It is common for these projects to require a mix of manual and machinery operations and different trench system configurations within a considerably small area. However, these challenges are compounded for sanitary upgrading projects because they typically require deeper excavation depths than other infrastructure upgrading projects. This is attributed to the following reasons: (1) the gravitational flow which results in increased depth as the network length increases (up to a certain point, after which a pump station is needed); (2) the depth of sewer lines has to be lower than water mains with predefined distances and in some cases specific pipe materials (where they are often co-located in slum areas due to the narrow and unplanned road network), which results in deeper excavation depths in possibly complicated structural conditions; and (3) the potentially random household sanitary outlet depths for buildings in the slum area. These issues require close attention to the excavation means and methods. To this end, the proposed framework dedicates two models for this issue, as explained in the “Resource Screening Model” and “Trench System Configuration Model” sections. Sixth, the proposed approach acknowledges the importance to allow for project-specific conditions that cannot be pre-defined for every sanitary upgrading project. These issues can include considerations for existing underground utilities or a need to prioritize service to areas of high need. As such, the model offers the flexibility to accommodate user-defined constraints in the form of recommended relationships, as described in detail in the “Network Prioritization Model” section.

It is noteworthy that some of aforementioned characteristics are not exclusive to sanitary upgrading projects in slum areas. For example, some characteristics such as road closures are also found in utility upgrading projects. The uniqueness of the sanitary upgrading projects in slum areas stems from the fact that it can encompass all of the aforementioned challenging characteristics and opportunities; and hence, it warrants a dedicated analysis and approach.

### Residents’ Resistance to Sanitary Upgrading Projects

Dwellers’ resistance to upgrading projects can stem from multiple reasons and can vary from project to project. Hence, any proposed approach to address this key issue needs to resolve the underlying reasons for resistance while being flexible to accommodate new approaches when working in different sites. The proposed methodology addresses the following underlying reasons that can contribute to or trigger dwellers’ resistance: (1) disruptions caused by earthwork operations and the associated road closure; (2) lack of clarity about the expected benefits from the upgrading project; and (3) the perceived unfairness on the order of service provision to various areas.

First, earthwork operations and the associated road closures can cause significant disruptions to the livelihood of residents in proximity to the construction sites. Some projects adopt an approach of mass excavation in multiple road segments, followed by pipelaying in all these segments, then backfilling, and so on. This approach, while less expensive, results in significant socioeconomic disruptions. Slums upgrading projects are very sensitive to these disruptions because of dwellers’ resistance. As such, the proposed framework requires completing the work on each road segment entirely (including excavation, pipelaying, backfilling, etc.). Even manhole installation is executed in parallel to other operations in the proposed approach. This approach is presented in the “Construction Time and Cost Analysis Model” section. The disruption to a specific area is also minimized by adopting a breadth-first search when prioritizing work on road segments. That approach ensures that work is conducted in the downstream segments of all areas before moving upstream in these areas. This is contrary to focusing operations on the downstream of one area and then moving upstream on the same area, which results in multiple road closures within the same area compounding the disruptions to dwellers of these areas. The breadth-first search forces the sequence of construction to move from one area to the other after completing a subset of the road segments and hance avoids intensifying the negative impacts on one area. The breadth-first search is explained in detail in the “Network Prioritization Model” section.

Second, residents often have doubts or lack clarity about the benefits expected from the construction work. These doubts will persist until tangible benefits are presented to nearby residents. Realizing the benefits of the upgrading project can turn resistance into excitement about and anticipation of the work to be completed. The proposed approach accelerates service delivery to downstream road segments by completing the sanitary upgrading work and turning on the services without the need to wait until construction work in the whole area is completed. That approach allows upstream residents in the same area to observe the benefits of the upgrading work downstream and appreciate the value to be delivered by the construction operations. Third, when residents are aware of the expected benefits, questions about who receives the services first can result in other types of resistance. In these cases, residents may try to exercise pressure to prioritize work in their areas before others. This is a question of fairness that is addressed by the model by the proposed breadth-first search. This approach provides services to all areas in parallel and as such eliminates the sense of unfairness or competition among the slum dwellers. On the other hand, if a depth-first search approach is adopted, all benefits will be accumulated in one area before moving to other areas. Such an approach can result in resentment among residents of areas where service is scheduled later and hence result in resistance from the locals. An essential aspect of the model is its flexibility to allow other considerations to be included in construction planning. This is made possible by defining mandatory and recommended priorities among road segments. Such modeling flexibility allows accommodating other factors that can mitigate resistance and that are unique to a specific slum area.

## Framework Implementation Models

The following subsections introduce each of the five implementation models. Each subsection is concluded by a statement that describes how the model addresses the logistical and/or resistance challenges associated with sanitary upgrading projects in slum areas.

### Resource Screening Model

A key success factor for slums upgrading projects is the ability to overcome unique construction and logistical challenges. One common challenge in slums is whether road segments will be accessible to available construction equipment. This is attributed to the unplanned and random nature of the road networks as well as their varying—and in many cases very narrow—road widths. The proposed resource screening model is designed to answer the following question: given the accessibility limitations within the slum area, which pieces of excavation equipment are suitable for the existing road network? It should be noted that in cases when no equipment can reach a specific road segment, manual excavation is always a possible solution, albeit an expensive and time-consuming one.

**Fig. 2:**
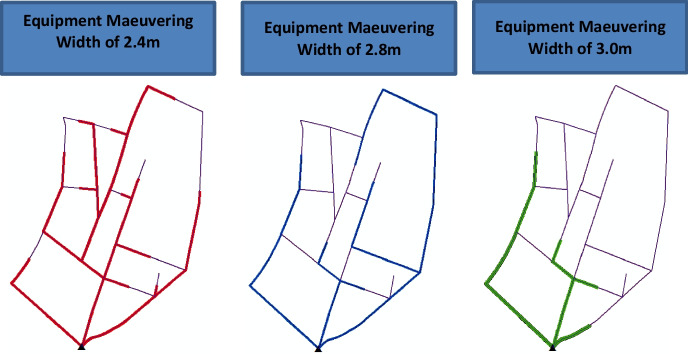
Thick lines: possible equipment routes associated with various equipment maneuvering widths. Fine lines: inaccessible segments to be manually excavated

The resource screening model takes into account existing road network characteristics, such as road segments’ widths and lengths as well as the existing external access points to the road network. The input also includes the maneuvering widths for the selected equipment set. The maneuvering width for an excavator is the smallest width to enable the excavator to move along the excavated trench. This model uses the maneuvering width instead of the equipment width in order to ensure that the equipment will avoid unnecessary back and forth maneuvering movements [28]. The resource screening model uses ArcGIS to perform two types of analyses. First, logistical analysis is performed to identify all possible equipment paths taking into consideration road and equipment characteristics. This is achieved using a shortest-widest route algorithm, as explained in more detail in this section. Second, access routing analysis is used to define equipment access points to the network. Applying these two analyses to each road segment, the model identifies (1) which equipment set can reach and work on that segment (as shown in Fig. 2) and (2) the shortest excavation path that each piece of equipment can take to reach the designated road segment. Figure 2 shows an example of running these analyses on an actual road network that was undergoing sanitary upgrading. Each alternative in the figure represents the possible road segments that a piece of equipment can access given the equipment maneuvering width.

The shortest-widest route algorithm finds the shortest path between a predefined starting point and end point using Dijkstra’s shortest path method “single-node shortest route problem” [29]. However, the algorithm adds to Dijkstra’s shortest path method the ability to account for a required minimum width for the selected path. In the proposed model, this minimum width is constrained to the equipment maneuvering width. ArcGIS’s Closest Facility Solver is used to perform the shortest-widest path computations. As described in this subsection, the resource screening model addresses some of the key logistical challenges associated with sanitary upgrading projects. These challenges include (1) the unplanned and random nature of the road networks where the sewer lines are located, (2) the varying and very narrow road widths, and (3) the limited accessibility of the road network.

### Trench System Configuration Model

The previous model identifies which road segments will use machinery (for a given equipment maneuvering width) and which will need to be manually excavated. The trench system configuration model then identifies the appropriate trench excavation system. The model answers the following construction question: Which trench excavation system is most suitable and most economic for each road segment? The model considers four commonly used systems for trench excavation, including vertical, sloped, battered, and shored excavation systems. The model employs user-defined criteria when processing possible alternatives in order to take into account structural and safety considerations as well as logistical considerations (such as each road width).

Input data to this model includes selected construction methods (manual or using equipment), sewer network design data (pipelines depths and diameters), road network attributes (road segments widths, soil characteristics, and groundwater table), and adjacent structures stability. The criteria for selecting the trench system varies depending on the construction codes in place for each country and locality. However, these codes typically use the above-mentioned input data. An application example representing an actual upgrading project is presented later in this paper. The criteria modeled in the trench system configuration model is customized to meet the requirements of the upgrading project used for the application example. A visual basic model is developed to assist with selecting the trench excavation system given these characteristics, where user can define the values for this input data. For projects in other locations, the criteria modeled in the trench system configuration model will need to be customized for their needs. The trench system configuration model addresses some of the unique logistical challenges associated with sanitary upgrading projects. These challenges stem from the varying excavation depths required to install the sewer lines based on gravitational flow considerations as well as underground road conditions. Furthermore, the model takes into consideration the potential structural stability issues for surrounding buildings.

### Construction Time and Cost Analysis Model

**Fig. 3:**
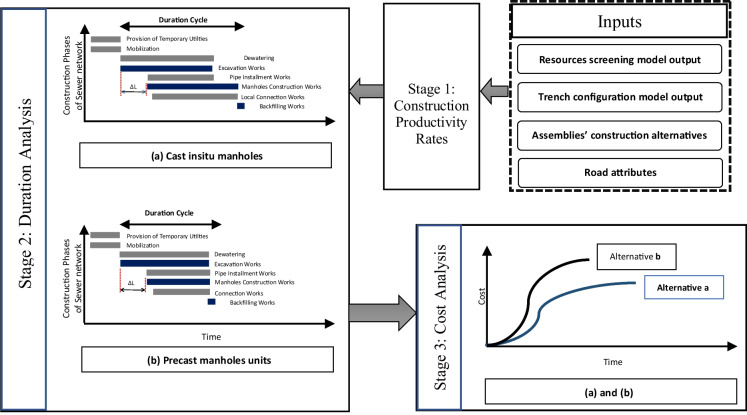
Proposed workflow of time and cost analysis

The third model in the framework is designed to answer the following question, as presented in Fig. 3: For each set of construction alternatives, what is the execution cost and duration for each road segment? To this end, the model computes construction time and cost by processing the following input: (1) sewer network design data (pipelines depths and diameters); (2) manholes construction assembly alternatives (either precast or cast-in-situ concrete); (3) key construction activities, such as excavation, pipe laying, manhole installation, the trench excavation system (as identified using the previous model), and trench backfilling; and (4) the productivity rates and unit costs associated with these construction activities.

There are two scenarios to be considered when estimating the duration of excavation and manholes installation in each road segment, as shown in Eq. 1. First, if the total duration of the road segment excavation is more than the duration of manholes installation throughout that segment, then the excavation work drives the critical path on that segment. This case is common when excavation is done manually while precast concrete is used for manhole construction. In this case, the total construction duration (∆D^*r*^) for a road segment *r* equals the summation of (1) the excavation duration (*ED*_*r*_) of road segment *r*; (2) the duration ($\Delta {D}_2^r$) needed to install two manholes in road segment *r*, which represent the first and last manholes in the segment; and (3) duration needed to lay the pipes, backfill, and rehabilitate the road segment (*Reh*. *D*_*r*_).

Second, if the total duration of road segment excavation is less than the duration of manholes installation throughout that segment, then the manhole installation drives the critical path. This case is common when machinery is used in excavation while cast-in-situ concrete is used for manhole construction. In this case, the total construction duration (∆D^*r*^) for a road segment *r* equals the summation of (1) the duration ($\Delta {D}_o^r$) needed to excavate the road segment *r* between the location of the first manhole and second manhole, which is a precondition to be able to work on the second manhole; (2) the manholes installation durations (*MD*_*r*_) throughout road segment *r*; and (3) duration needed to lay the pipes, backfill, and rehabilitate the road segment (*Reh*. *D*_*r*_).

1 $$\Delta {\textrm{D}}^r=\left\{\begin{array}{c}{ED}_r+\Delta {D}_2^r+ Reh.{D}_r\kern3em \textrm{IF},\kern1em {ED}_r\ge {MD}_r\\ {}\ \\ {}{MD}_r+\Delta {D}_o^r+ Reh.{D}_r\kern3em \textrm{IF},\kern1em {ED}_r<{MD}_r\kern0.5em \\ {}\kern3.5em \end{array}\right.$$

Equation 1 assumes that excavation work and manhole installation can overlap within the same road segment as long as the work does not overlap at the same location within the road segment. This assumption is practical and has been observed in the case study presented later in this paper. It was selected in the proposed model because it accelerates the construction work and hence reduces the period of road closure and associated disruptions to residents. If a construction planner decides to complete earthwork before starting manhole installation, then Eq. 1 would need to be revised to add (1) the total duration needed for excavation (*ED*_*r*_); (2) the total duration for installing all the manholes within the road segment (*MD*_*r*_); and (3) duration needed to lay the pipes, backfill, and rehabilitate the road segment (*Reh*. *D*_*r*_).

**Fig. 4:**
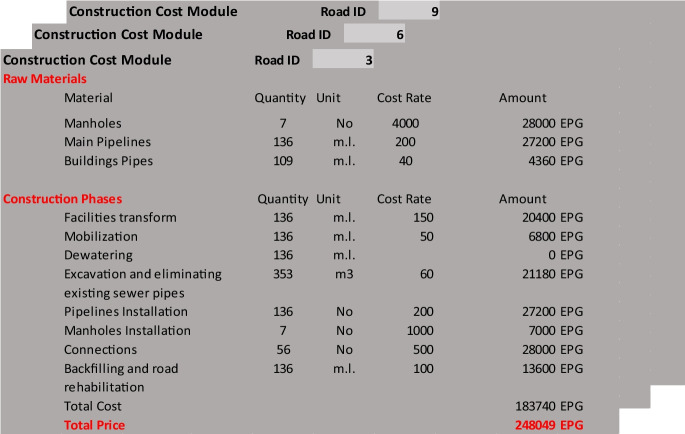
A screen capture of the time and cost analysis model

As shown in Fig. 4, the model enables the user to define the construction costs of each road segment including the pipeline to be installed and manholes. The user can also define the cost of mobilization and provision of temporary utilities. The input to this model builds on the excavation method and equipment determined by the resources screening model and the trench configuration system. The proposed workflow of the time and cost application is presented in Fig. 3. Whereas Fig. 4 shows a screen capture of applying the model on an actual project site. Using the information entered to the model (as shown in Fig. 4), the model computes the upgrading costs for each road segment by multiplying the road segment work quantities to the user-defined unit cost rates. The total upgrading cost includes adding the cost of mobilization and temporary utilities.

As explained in this subsection, the construction time and cost analysis model is designed based on assumptions of an accelerated construction sequence. This is essential to minimize the disruptions and periods of road closure resulting from the upgrading work and to accelerate the provision of sanitary services to the slum dwellers. Both outcomes contribute to mitigating the dwellers’ resistance to the upgrading work.

### Network Prioritization Model

For any sanitation network upgrading project, a large number of possible construction plans may exist. This model is developed to identify possible construction sequences among road segments. It answers the following question: What are the possible construction sequences for sanitary upgrading in a specific road network? The priority and sequence of upgrading and delivering the sanitation services in a proposed network can be controlled by two types of relationships, namely, mandatory relationships and recommended relationships. These relationships inform decision-making and allow organizing the scope of work into priority packages. Accordingly, upgrading work for road segments in the higher priority package should be executed before road segments in a lower priority package. On the other hand, road segments sharing the same priority package can be organized under various upgrading sequences representing the possible combinations of work.

A mandatory relationship should not be violated and is governed by two criteria. First, the flow direction, where the outlet pipeline of each manhole is required to be finished before the manhole inlet pipelines. Second, work propagation technique, where the breadth-first search (BFS) technique is adopted. Breadth-first search is selected because it allows the construction plan to achieve two important objectives which are road segments and upgrading sanitary infrastructure. Road segments in the same level usually have similar widths and hence require similar pipe diameters and use the same excavation equipment. Accordingly, working on these similar road segments at the same time is more practical from constructability and logistical points of view and usually results in a more time-efficient and cost-efficient operation. Upgrading sanitary infrastructure in road segments at the same level provides services to multiple areas simultaneously, which is an important element in ensuring residents’ satisfaction and a means to mitigate possible public resistance to upgrading work. Residents’ resistance can have detrimental effects on the success of the upgrading project in slum areas. Therefore, breadth-first search offers a sequence which is more practical because it allows progress in all areas to avoid residents’ resentment and resistance.

**Fig. 5:**
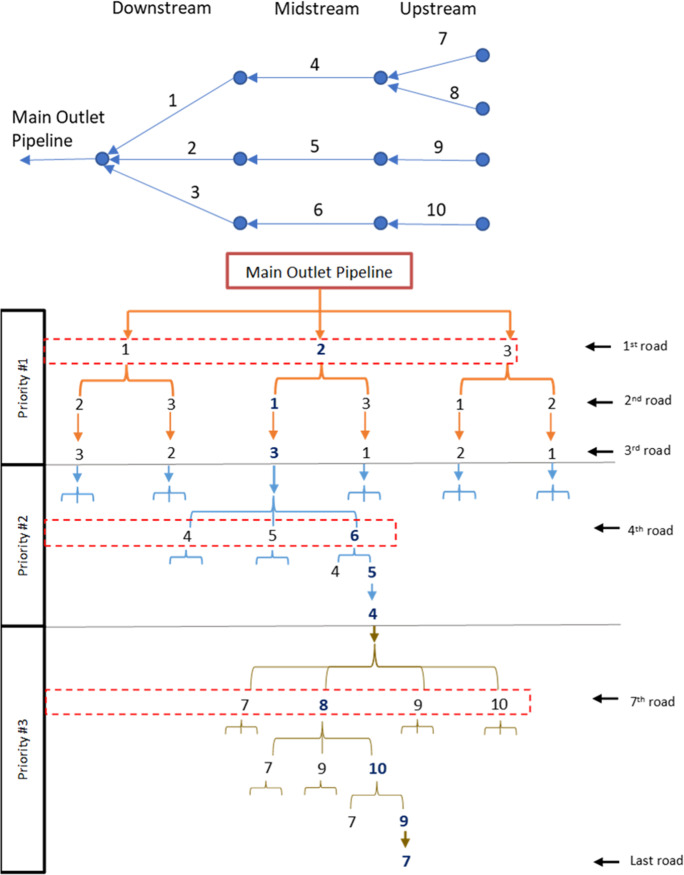
Example of possible network prioritization schemes

Figure 5 is used to demonstrate how mandatory relationships and priority packages are determined using the two criteria of flow direction and breadth-first search. In Fig. 5, the arrows represent the road segments where the sewer pipelines are located, and the arrow heads point to the flow direction. As shown, road segments 1, 2, and 3 are the downstream segments in the network, so the breadth-first search groups them under priority package #1. A mandatory relationship is created to require that upgrading work proceeds in these three segments first. Second, road segments 4, 5, and 6 are midstream, so the breadth-first search groups them under priority package #2. Similarly, a mandatory relationship is created to require that upgrading work proceeds in these three segments second. Lastly, road segments 7, 8, 9, and 10 are the upstream segments in the network, so the breadth-first search groups them under priority package #3. A mandatory relationship is created to require that upgrading work proceeds in these four segments last. As such, the network prioritization model organizes the road segments in the network into three priority packages based on flow direction and breadth-first search.

A recommended relationship, on the other hand, is used to recommend an internal sequence for executing upgrading work among road segments sharing the same priority package. This is an optional relationship used to increase the efficiency of the upgrading strategy based on conditions that are unique to each project site. Three examples of unique project conditions are presented to demonstrate the value of the recommended relationships. In the first example, different sequences of road closures could result in different levels of traffic impacts. Depending on these impacts, recommended relationships could be used to enforce executing the sequence of work with the least impact on traffic. This is a very important consideration for sanitary upgrading projects. In the second example, a specific sequence of work could be preferred to avoid field conditions in a specific route (such as the existence of underground cables or oil and gas pipelines). In this example, a recommended relationship can guide the construction sequence to start execution in a road segment free of utility interreferences and allow more time to resolve utility conflicts in another road segment. With this insight regarding subsurface conditions, an improved execution plan can be implemented. Hence, offering that flexibility in defining relationships is valuable to overcome logistical challenges that are site-specific and cannot be pre-defined in the model for all projects. In the third example, a participatory planning approach could be adopted in which residents work with the planning team to identify the sequence of work that minimize socioeconomic disruptions while accelerating service provision to areas in high need. As such, a specific sequence of work would be recommended to implement the agreed-on plan with residents. For example, road segments 1, 2, and 3 share the same priority package as shown in Fig. 5. If residents in road segment 2 completely lack access to the sewer network, whereas residents in road segments 1 and 3 need repairs to their existing sewer lines, then a recommended relationship can be defined to prioritize service to residents in road segment 2. That sequence of work will be enforced in the model by introducing user-defined relationships. Such modeling flexibility is essential to minimize disruptions to slum dwellers and mitigate possible resistance. The network prioritization model requires the following input from the users: (1) flow direction as per the sewer network design, which defines mandatory sequencing, and (2) the user-defined recommended priorities among some segments based on need.

This hierarchy of priority packages not only offers modeling capabilities, but it also introduces computational efficiencies. It reduces the search space of the sequencing problem by eliminating impractical sequences of work. For example, for the small network of 10 road segments presented in Fig. 5, there can be 3,628,800 (or 10!) possible prioritization schemes. Let us assume that these 10 segments can be organized in three priority packages: segments 1-3 in priority package #1, segments 4-6 in priority package #2, and segments 7-10 in priority package #3 (as shown in Fig. 5). In this case, the search space is reduced to 864 (or 3! × 3! × 4!) possible prioritization schemes only. This eliminates more than 3.6 million impractical sequences representing 99.98% of the search space. Figure 5 shows examples of different prioritization schemes for these 10 road segments prioritized into three priority packages, where the flow of each branch represents the order of road completion.

Furthermore, the network prioritization model employs a network reduction technique. A large number of road segments will result in a very large number of prioritization schemes. In order to reduce the search space and achieve a computationally efficient model, a network reduction process is introduced. This process combines road segments where manual excavation will take place with preceding segments where excavation equipment will be used. This combination is only for analysis purposes and does not affect the selected excavation method for each segment. These segments are combined because segments with manual excavation can always be excavated while their preceding segments are being excavated using equipment since different crews are executing the work. Hence, there is no value in including the manually excavated segments separately in the prioritization process. Combining these road segments is accompanied by aggregating two key attributes for these segments. First, the number of served population is aggregated to represent an accurate count for the combined segments, as shown in Eq. 2. And second, the cost of upgrading the combined segments is also aggregated to reflect the total cost of upgrading a preceding segment using equipment and its succeeding segment (or segments) using manual labor. After adjusting the number of segments, a permutation algorithm is used to generate all proposed prioritization schemes [30]. This algorithm is run on each priority package P.

2 $$\Delta {\textrm{S}}^r=\Delta {S}_0^r+\sum_{j=1}^n\left(\Delta {S}^j\right)$$

3 $$\Delta {\textrm{C}}^r=\Delta {C}_0^r+\sum_{j=1}^n\left(\Delta {C}^j\right)$$

where ΔS^r^ and ΔC^r^ ΔC^r^refer to the adjusted values of served population and construction cost for a road segment *r*, respectively, after combining succeeding segment(s); $\Delta {S}_0^r$ $\Delta {\textrm{S}}_0^{\textrm{r}}$and $\Delta {C}_0^r$ refer to the served population and construction cost for road segment r, respectively, before combining succeeding segment(s); ΔS^*j*^ and ΔC^*j*^ refer to the served population and construction cost for road segment *j*, respectively, where these segments will be excavated manually and the number of these succeeding segments is *n*.

As shown in this subsection, the network prioritization model plays a key role in addressing both logistical and resistance challenges. The mandatory relationships accelerate service delivery to downstream road segments using mandatory relationships and distribute the service provision among the various sections within the slum area using the breadth-first search. Both aspects are important to mitigate resistance and to showcase the value of the upgrading work to slum dwellers. The recommended relationships, on the other hand, provide the needed flexibility to account for project-specific logistical constraints (such as underground utilities) and resistance challenges (such as the importance of prioritizing service to areas of high need). The role of recommended relationships in the model is limited to defining the sequence of work among segments sharing the same a priority package. This modeling features balances the amount of flexibility to allow the model to serve as a practical tool that could be used by planners without overcomplicating the modeling requirements.

### Serviceability and Cost Assessment Model

When the upgrading project is completed, all households in the served area will benefit from the sanitary services. However, sanitary services can be turned on for some residents before the completion of the whole project if sanitary infrastructure in their road segment is upgraded and if downstream segments are also upgraded allowing the flow of sewage uninterrupted until the network outlet. Accordingly, optimal construction plans and their prioritization schemes can result in faster service delivery to residents compared to other plans. In order to identify these optimal plans, there is a need to assess the serviceability performance of various plans using an objective measure.

**Fig. 6:**
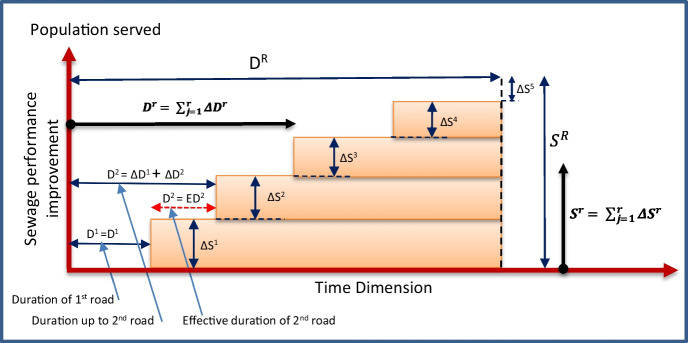
Serviceability performance analysis

The proposed framework introduces a novel approach to select the construction sequence that accelerates service delivery to slum residents. This is achieved by answering the following research question: How to assess the serviceability performance of each proposed construction plan? To this end, the serviceability and cost assessment model introduces a serviceability index to represent how fast sanitary services are provided to each group of households in the served area. This concept is explained in detail in this subsection. When completing the upgrade of the sanitary infrastructure in road segment *r*, then a number of residents ΔS^r^ can benefit from the sanitary services if the downstream segments are also upgraded. In this discussion, we will assume that a proper sequence of work is selected to guarantee that downstream segments are always served first, since this is already achieved using mandatory relationships as explained in the previous model. By the completion of upgrades in segment *r*, the total number of served households (S^*r*^) from the start of the project equals ΔS^*r*^ in addition to the served households in previously upgraded segments, as presented in Eq. 4 and Fig. 6. The total number of served households (S^*R*^) by the upgrading project can be calculated using Eq. 5, where *R* is the total number of upgraded network segments.

4 $${S}^r=\sum_{i=1}^r{\varDelta S}^i,i=1,2,\dots, r$$

5 $${S}^R=\sum_{i=1}^R{\varDelta S}^i,i=1,2,\dots, R$$

The duration of upgrading the sanitary infrastructure in road segment *r* is ΔD^*r*^. However, the total time duration (D^*r*^) needed to upgrade segment r from the start of the project equals ΔD^*r*^ in addition to the time needed to upgrade preceding road segments, as presented in Eq. 6 and Fig. 6. Similarly, the total duration of the upgrading project (D^*R*^) is calculated using Eq. 7.

6 $${D}^r=\sum_{i=1}^r{\varDelta D}^i,i=1,2,\dots, r$$

7 $${D}^R=\sum_{i=1}^R{\varDelta D}^i,i=1,2,\dots, R$$

By the completion of upgrades in segment *r*, the served households ΔS^*r*^ will benefit from the sanitary services until the completion of the upgrading project and will continue to benefit afterwards. Therefore, the added benefit to this group of households can be calculated by multiplying the number of served households ΔS^*r*^ by the duration of service until the project completion, which is function in the selected construction sequence. This duration is equal to the total duration of the project D^*R*^ minus the total duration (D^*r*^) needed to upgrade segment *r* from the project start date, as shown in Fig. 6. Accordingly, a serviceability index (SI) can be calculated for all households in the serviced area using Eq. 8. The higher the value of SI, the longer the duration that households are being served by sanitary upgrades. This translates to a faster delivery of services to residents.

8 $$SI=\sum_{r=1}^R\ \left\{{\varDelta S}^r\ x\ \left({D}^R-{D}^r\right)\right\},r=1,2,\dots .,R$$

where *D*^*R*^ is the total duration of sanitation network upgrading project, D^*r*^ is the cumulative upgrading duration calculated from project start to completing upgrading work for road segment 𝑟, *ΔS*^*r*^ represents the number of residents receiving the benefit of the upgrading work in each road segment *r*, and *R* represents the total number of roads. It is noteworthy to mention that SI can be graphically represented by the shaded area under the curve in Fig. 6. A larger area represents an accelerated delivery of sanitation services to residents. The area under the curve represents the multiplication of the number of residents (population) benefiting from the upgraded sanitary services by the duration (in days) during which they received the upgraded services before waiting for the project completion. Hence, the unit of measurement of SI is pop.days (i.e., population days).

After selecting the trench excavation system and construction equipment, the total construction cost can be computed as previously presented in the “Construction Time and Cost Analysis Model” section. Different construction plans and prioritization schemes will share the same total cost but will have different rates of expenditures resulting in different cashflow curves. Considering the time value of money, each construction plan will have a unique net present value for its total cost, which should be accounted for when selecting the optimal plan. Equation 9 computes a cost index (CI) representing the net present value of the total cost, where ΔC^r^ is the upgrading cost of road 𝑟 and u is the interest rate. That interest rate should account for the combined effect of general inflation as well as the specific price escalation relevant to construction costs. The total upgrading cost includes adding the cost of mobilization and temporary utilities (*C*_*o*_).

9 $$CI=\sum_{r=1}^R\frac{{\varDelta C}^r}{\ {\left(1+u\right)}^{D^r}}+{C}_o,r=1,2,\dots .,R$$

It should be noted that in order to have a complete picture of the construction costs, a construction cost contingency should be computed by conducting a risk assessment exercise. This assessment would quantify the risk exposure for each construction site (including, for instance, exposure to unforeseen underground conditions). The model currently does not include a risk assessment module, which can be part of future work developments. It is also noteworthy to mention that an opportunity cost could be added to the total cost to quantify the impacts of dwellers’ resistance on the project execution. However, the serviceability index is introduced to objectively assess the ability of the proposed construction plan on accelerating service delivery to mitigate dwellers’ resistance. Therefore, introducing an opportunity cost to quantify resistance would result in double counting the impact of resistance when optimizing construction plans. For example, if a construction sequence is identified that could reduce the time *D*^*r*^ to provide sanitary services for some residents by one month, then the Serviceability Index would increase as demonstrated using Eq. 8. Accordingly, the resistance of these households and their surrounding households to the upgrading project work would decrease (or even stop) because of their realization of the value the project brings to their livelihoods. Accordingly, the increase of value in the serviceability index corresponds to a decrease in resistance. As such, serviceability index is used in lieu of adding an opportunity cost for the reduced resistance.

As shown, the presented framework and its key models support construction planners in selecting the most suitable construction approach (trench excavation system and construction equipment) and presents a range of possible prioritization schemes. For each scheme, this framework computes a SI and CI. The next step is to identify the optimal construction plan and prioritization scheme that can maximize SI while minimizing CI. To this end, a multi-objective optimization model is developed and is presented in the next section.

## Optimization Model

A multi-objective optimization model is developed to identify the optimal sanitation upgrading strategies in order to accelerate the benefits delivered to residents while controlling the upgrading construction costs. The following subsections present the model formulation and implementation.

### Model Formulation

**Fig. 7:**
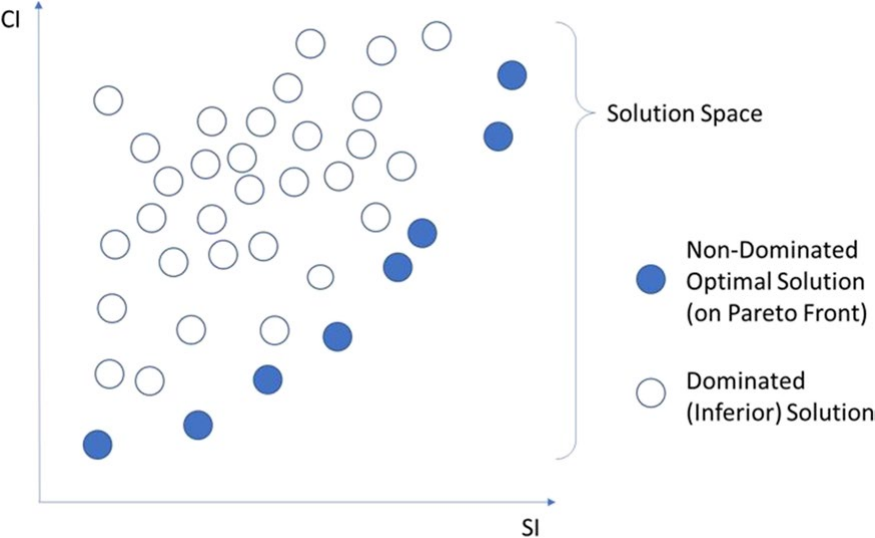
An illustration of a solution space and the Pareto front

There are two optimization objectives in the proposed model: (1) maximizing the SI to accelerate service delivery and (2) minimizing the cost index representing the net present value of the upgrading costs (CI). Each prioritizations scheme will result in a unique SI (as computed using Eq. 8) and CI (as computed using Eq. 9). To this end, the goal of the optimization model is to generate the Pareto front representing the optimal tradeoffs between these two objectives. Figure 7 illustrates the concept of Pareto optimal solutions, which are the solutions that dominate all other solutions in the search space in at least one of the optimization objectives.

In the proposed optimization model, decision variables are designed to represent the possible prioritization schemes for executing the upgrading project. As demonstrated in the network prioritization model, the road segments in a network can be classified under a number of priority packages based on mandatory relationships. Furthermore, road segments in these priority packages can be sub-classified based on recommended relationships. These sets of mandatory and recommended relationships reduce the search space significantly by maintaining only practical execution plans for further investigation. As such, an optimization model is needed to prioritize the execution of upgrading work in road segments sharing the same priority package or sub-package. To further reduce the search space, and as explained in the network prioritization model, road segments with manual excavation will be combined in the decision-making process with preceding road segments where excavation equipment is needed. This step reduces the number of alternatives to be represented by each decision variable.

Employing this modeling approach, there can be N decision variables (X1, X2, …, XN). Each decision variable Xn represents the possible permutations of executing upgrading work in road segments sharing the same priority package (Pi) or sub-package (Pi-Rj); where there is a total of *I* priority packages (for every *i* = 1, 2, …, *I*); and within each package, there can be up to *J* sub-packages based on recommended relationships (for every *j* = 1, 2, …, *J*). A set of possible values to the decision variables represent a viable prioritization scheme. Considering all possible permutations of decision variables values represent the search space of a total of *S* prioritization schemes.

**Fig. 8:**
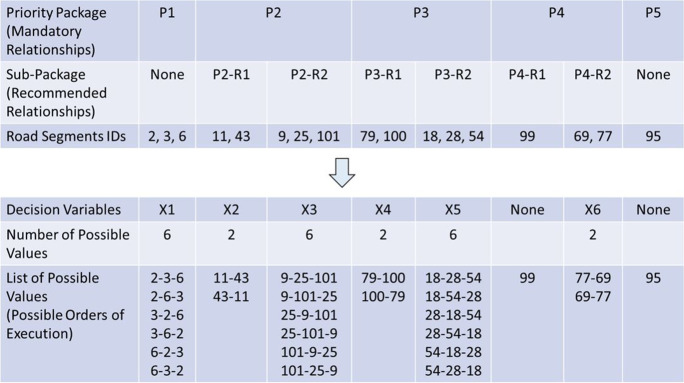
Enlisting priority packages and decision variable information

To better understand the decision variables definition, an illustrative example is presented, as shown in Fig. 8. This example is based on an actual upgrading project and will be further analyzed in the following “Application Example” section. In this network, there are 28 road segments. However, after combining manually excavated road segments with preceding segments where excavation equipment will be used, the network is reduced to 17 segments. These segments are classified under five priority packages, as shown in Fig. 8. For instance, priority package P1 includes the road segments with IDs 2, 3, and 6. As discussed in the “Network Prioritization Model” section, the decisions on the number of priority packages and which road segments should be included within each priority package depend on the flow direction and breadth-first search. Road segments in the downstream of the network are grouped under the first priority package. This creates a mandatory relationship that sanitary upgrading work for these downstream segments must completed first before other segments in the network. Based on flow direction, road segments with IDs 2, 3, and 6 are the downstream segments and hence are grouped under priority package #1. Once work is completed on these segments, service can be turned on while construction work is in progress in the rest of the network. Using flow direction and breadth-first search, the model identifies that the immediate upstream segments from the segments in priority package #1 are road segments with IDs 9, 11, 25, 43, 101. These five segments are grouped under priority package #2. Following this procedure, the model identifies a total of five priority packages for the 17 road segments in the network, where roads segments in each priority package are the immediate upstream segments from the preceding priority package based on the flow direction. That approach accelerates benefits realization to the residents in downstream which is important to showcase the value that the upgrading project brings to the slum dwellers. As discussed earlier, the early benefits realization by some residents mitigates possible resistance to the upgrading projects.

Some priority packages are further broken down into sub-packages based on a number of recommended relationships. For example, priority package P2 is broken down into two sub-packages: P2-R1 and P2-R2. Sub-package P2-R1 includes two road segments (with IDs 11 and 43) and P2-R2 includes three road segments (with IDs 9, 25, and 101). Figure 8 lists all priority packages and sub-packages and their associated road segments. Decision variables are defined to represent the possible permutations within these packages and sub-packages. For example, decision variable X1 has six possible values representing all possible permutations to the execution order of road segments 2, 3, and 6 within priority package P1. These permutations include the following execution orders; 2-3-6, 2-6-3, …, 6-3-2. Similarly, decision variable X2 has two possible values representing possible permutations to the execution order of road segments 11 and 43 within priority sub-package P2-R1. As shown in Fig. 8, there are six decision variables, where sub-package P4-R1 does not need a decision variable since it only includes one road segment as well as priority package P5 which also includes one road segment. At the level of the main priority packages, it should be noted that cross-package permutations are not allowed in this model because it violates mandatory relationships. For example, a permutation that orders road segments from priority package #2 before segments from priority package #1 violates the mandatory relationship that all segments in a higher priority package must be completed before segments in lower priority packages.

Based on the possible values of these six decision variables, there are 1728 possible prioritization schemes for this upgrading project, all of which meet the mandatory and recommended execution relationships. These 1728 prioritization schemes represent the search space for this optimization problem and are generated by considering all possible permutations for the decision variables (i.e., 1,728 = 6 × 2 × 6 × 2 × 6 × 2). For example, one possible prioritization scheme is to execute upgrading work in road segments in the following order: (2-3-6) – (11-43) – (9-25-101) – (79-100) – (18-28-54) – 99 – (77-69) – 95, where road segments between parentheses share the same priority package or sub-package and their order of execution is represented using one decision variable, and road segments not between parentheses are not represented using a decision variable because they do not share a package or sub-package with other road segments.

### Model Implementation

Given the nature of this optimization problem and its formulation, multiple optimization techniques could be used. In this research, multi-objective genetic algorithms (MOGA) are used for the following reasons: (1) its effectiveness and computational efficiency in optimizing problems of this size and nature; (2) its modeling flexibility, which allows expanding the model in future work; and (3) its ability to optimize multiple objectives generating a Pareto front without requiring combining the objectives in a single function. Specifically, non-dominated sorting genetic algorithms II (NSGAII) is adopted in the optimization model implementation because of its high performance. This is attributed to the algorithm’s exceptional characteristics, such as quick non-dominated sorting, crowding, and elitism [31].

## Application Example

**Fig. 9:**
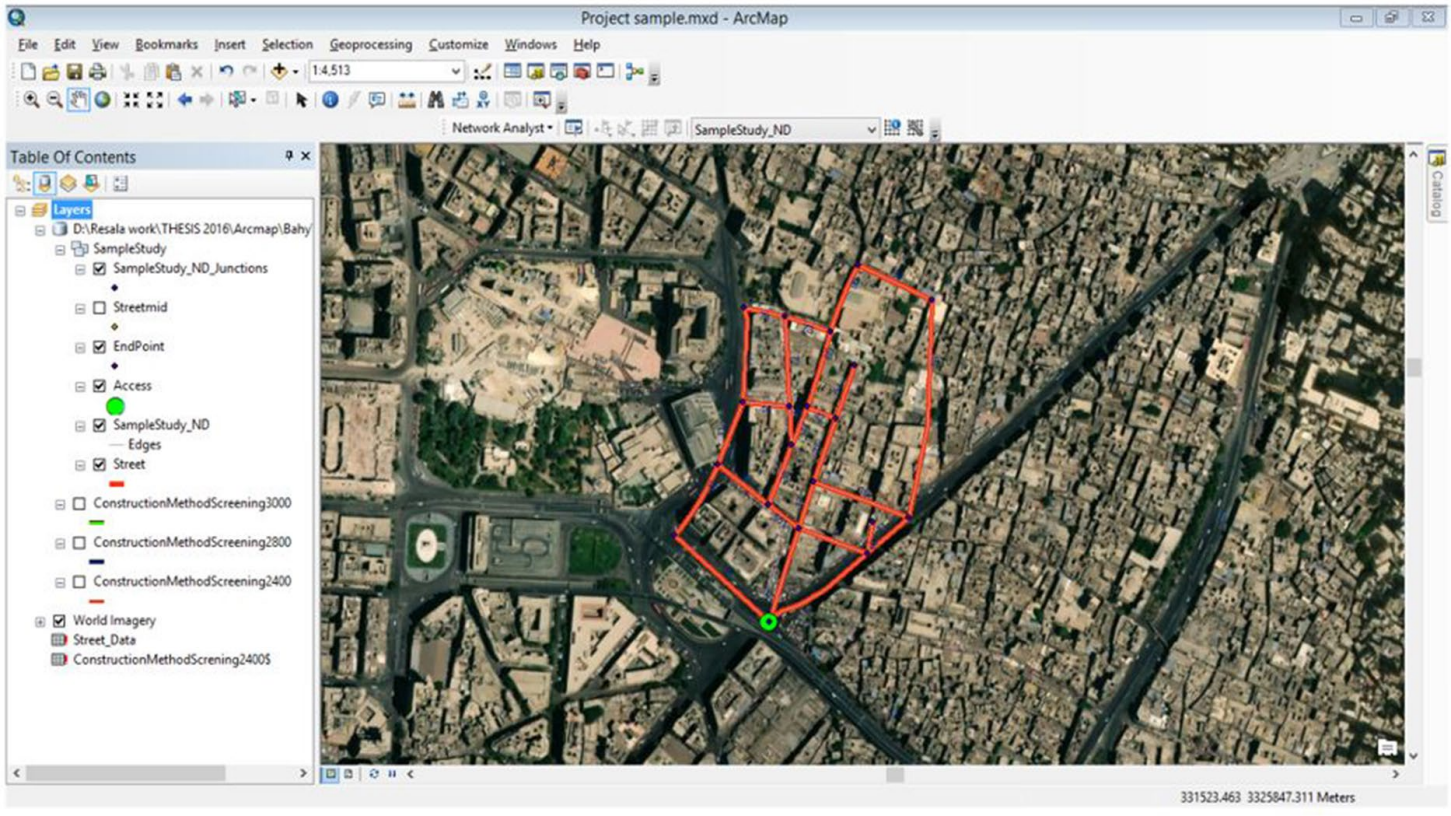
Satellite image of the project location, where the red lines represent the road segments subject to sanitary upgrading

An application example is presented in order to demonstrate the proposed model capabilities and to discuss the optimization results and model limitations. The application example represents an actual sanitation upgrading project that started construction in 2015 in the Azhar district in Cairo, Egypt (as shown in Fig. 9). The upgrading project serves about 9000 households (about 36,000 people) residing in an area of 270 m × 550 m (about 150,000 m^2^), which is mostly unplanned and densely populated. The scope of work includes the earthwork and installation of 2.2 km of sewer pipelines (for a total of 27 pipelines with various diameters) and associated manholes. To provide more detail about the scope of the sanitary upgrading project, Table 1 lists the number of households to benefit from the sanitary upgrading works in each road segment. The proposed framework is implemented on this application example including its five key models; the resources screening model, trench system configuration model, construction time and cost analyses model, network prioritization model, and the serviceability and cost assessment model, as shown in Fig. 1 and as summarized in the following subsections. Furthermore, the optimization model is used to optimize the execution plan.

**Table 1:** Network attributes

Road (*r*)	Served population (households)	Construction duration (days)	Construction cost (EPG)	Priority package “P”	Sub-package “R”
2	600	16	238,788	1	-
3	725	16	281,068		
6	675	13	226,980		
11	275	12	102,180	2	1
43	700	14	208,973		
9	350	11	78,137		2
25	475	10	121,631		
101	175	7	57,598		
79	1,000	28	362,740	3	1
100	525	10	162,988		
18	475	14	108,174		2
28	1,100	11	187,604		
54	275	9	58,866		
99	400	11	109,457	4	1
69	375	11	82,839		2
77	875	12	283,545		
95	325	10	75,432	5	1

### Framework Application

**Fig. 10:**
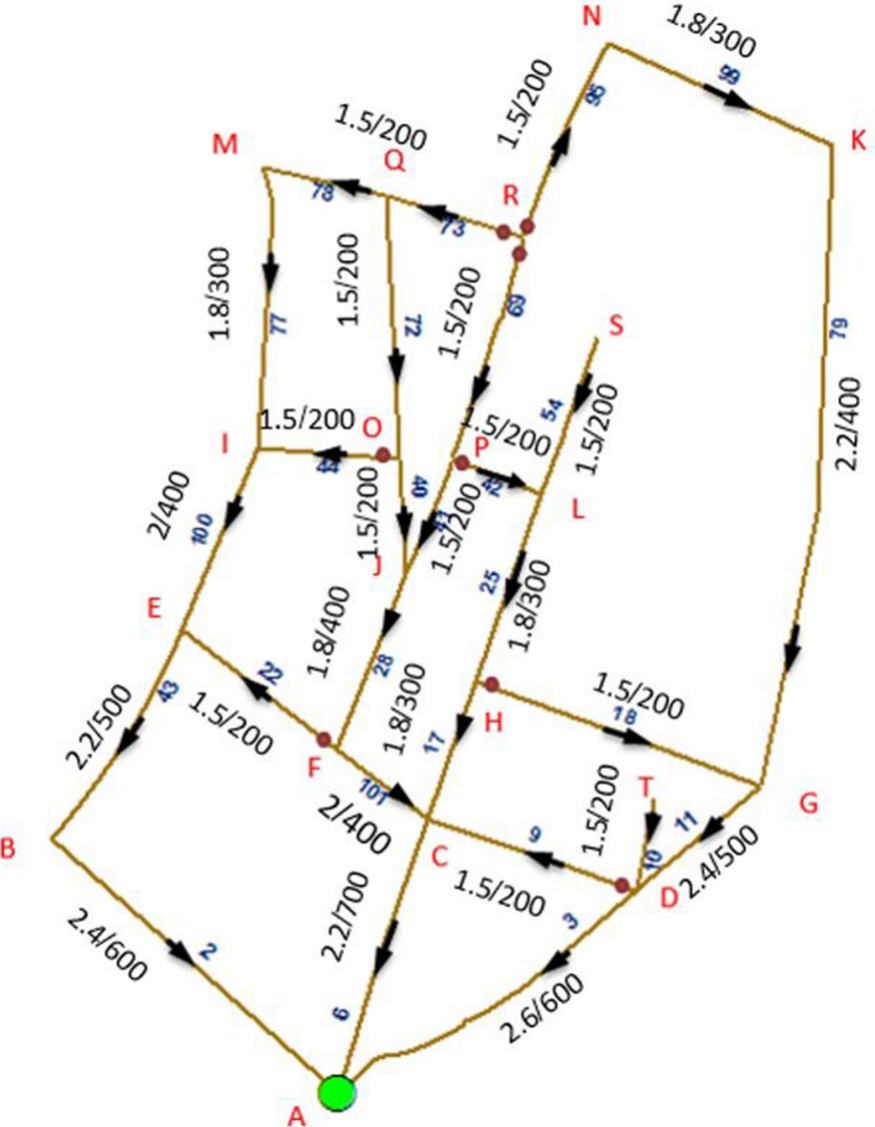
Sanitary network design attributes (represented as depth (m)/pipe diameter (mm); arrows denote flow direction)

First, the resource screening model was used to identify which road segments can be served by the contractor’s equipment and which will be manually excavated. The equipment used had a maneuvering width of 2.8 m, and hence, the model identified the proper access point to the network and the segments to be served, as shown in Fig. 10. Second, the trench system configuration model was used to identify the suitable trench system. Given the non-cohesive soil characteristics of the served area and the finding that the surrounding structures will not be affected by dynamic vibration, various trench systems were selected for this upgrading project. Selected trench excavation systems included vertical excavation with shoring system, vertical excavation without shoring, and tapered excavation. The criteria mentioned in the “Trench System Configuration Model” section is applied to this application example. For example, for a road segment where the adjacent structures are stable, the soil nature is cohesive, and the required excavation depth ranges between 1 and 1.5 m, the model selects vertical excavation. In another segment that shares the same soil characteristics and required excavation depth, but the stability of the surrounding structures is questionable, then a shoring system is selected.

Third, the construction time and cost analyses model is used to compute the construction cost and duration associated with each road segment in the upgraded network. Table 1 shows the results of this analysis. Fourth, the network prioritization model is used to generate all possible prioritization schemes. To this end, manually excavated segments are first combined with preceding segments where excavation equipment will be used. This resulted in a total of 17 road segments to be prioritized. Then, mandatory relationships were defined to ensure that outlet pipelines are executed before inlet pipelines. This information was used to categorize road segments into five priority packages. Moreover, construction conditions and associated traffic closures were used to define recommended relationships, which were used to breakdown priority packages P2, P3, and P4 into sub-packages. For each one of the 17 road segments, Table 1 lists the road segments IDs, population served, construction duration, construction cost, priority package, and sub-package. This information is processed by the model to enumerate all possible execution orders within each priority package and sub-package, as shown in Fig. 8. Fifth, the serviceability and cost assessment model together with the multi-objective optimization model are used to (1) compute the serviceability index (SI) and CI for each possible prioritization scheme; and (2) identify the Pareto front within the search space. This Pareto front represents the prioritization schemes that offer the optimal tradeoffs between SI and CI. Figure 8 shows the six decision variables representing this optimization problem and used to identify the optimal solutions among the 1728 possible prioritization schemes for this upgrading project.

**Fig. 11:**
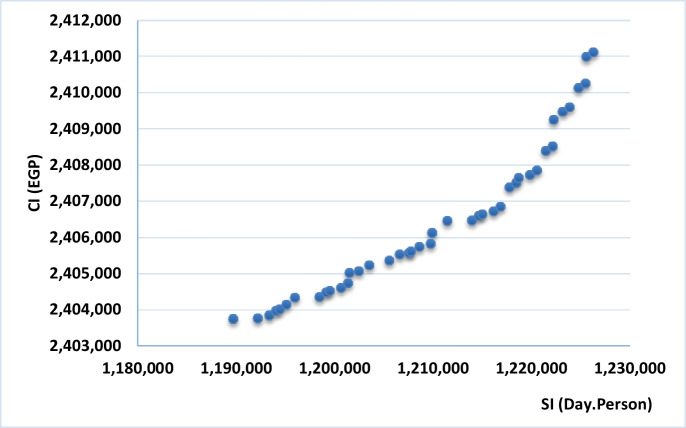
Generated Pareto front representing optimal tradeoffs between SI and CI

Before running the optimization model, a number of parameters are set. An interest rate of 25% per year is considered for the application example. As discussed before, this user-defined rate accounts for the general inflation rate as well as construction specific escalation rate. Genetic algorithms parameters in NSGAII were set to use a population size of 100, a crossover rate of 0.9, and a mutation rate of 0.005. Figure 11 shows the optimal tradeoffs between maximizing SI and minimizing CI after running the model for 1000 generations.

### Results and Discussion

Figure 11 shows the 42 optimal solutions generated by the optimization model. Each point represents a viable prioritization scheme that offers an optimal tradeoff between maximizing SI and minimizing CI. Each solution is feasible since each solution meets all mandatory and recommended relationships. Table 2 provides the details of six of the 42 optimal solutions; where ID represents the identification number of each of the generated solutions, and X1 to X6 represent the selected values for each of the six decision variables. For example, and as previously shown in Fig. 8, decision variable X1 has six possible values representing the six possible permutations of road segments orders of execution. These six permutations are 2-3-6, 2-6-3, …, 6-3-2. The solution with ID #1 includes selecting the second value for X1, which corresponds to executing the road segments in the order of 2-6-3. Considering all the selected values for decision variables X1 to X6, the identified optimal solution for solution #1 requires executing the upgrades in the road segments with the following order: (2-6-3)-(11-43)-(9-101-25)-(79-100)-(54-18-28)-99-(69-77). The road segments between parenthesis represent segments within the same priority package and sub-package and their permutation is modeled using one decision variable. Road segment #99 is the only segment in sub-package P4-R1, and hence is not represented with a decision variable since there are no permutations to select from.

**Table 2:** Pareto front optimal solutions

ID	X1	X2	X3	X4	X5	X6	X7	Serviceability index (day person)	Cost index (EGP)
**1**	2	1	2	1	5	1	2	1,189,700	2,403,754
**2**	1	1	2	1	5	1	2	1,192,200	2,403,770
**3**	1	1	5	1	5	1	2	1,193,350	2,403,852
**…**	…	…	…	…	…	…	…	…	…
**40**	3	2	4	1	3	1	1	1,225,450	2,410,255
**41**	3	2	4	2	4	1	1	1,225,550	2,410,987
**42**	3	2	4	2	3	1	1	1,226,250	2,411,111

It is noteworthy that the range of values on the Pareto front is not large, as shown in Fig. 11. The difference between the highest and lowest SI values on the Pareto front is 3%, and the difference between the highest and lowest CI values on the Pareto front is only 0.3%. As such, and from a practical standpoint, all 42 optimal solutions offer comparable SI and CI performances. Decision makers may select the solution with highest SI since the difference in CI is only 0.3%. Alternatively, decision makers may elect to consider additional factors to select among these optimal solutions, such as site layout considerations, construction risk, and political or public interest in selecting solutions that allow accelerating service delivery to specific areas. Such criteria are project specific and not modeled in the proposed generic model. There are other means that could be used to select among the identified optimal solutions. A host of commonly used approaches rely on defining weights for each optimization objective and then formulating a single objective function based on the weighted objectives. For example, Equation 10 develops a single-objective function by normalizing the serviceability and cost objectives over the identified Pareto front and employs a weighted average approach to identify the optimal solution. Nevertheless, given the close range of values for both SI and CI among the Pareto front in this application example, offering a weighted approach to compute a single objective function will not add much practical value to decision makers.

10 $$\mathit{\min}\kern0.62em Obj={W}_{SI}\times \frac{\left( SI-S{I}_{min}\right)}{\left(S{I}_{max}-S{I}_{min}\right)}+{W}_{CI}\times \frac{\left( CI-C{I}_{min}\right)}{\left(C{I}_{max}-C{I}_{min}\right)}$$

where Obj is the value of the single objective function to be minimized, WSI and WCI are the relative weights of SI and CI, SI_min_ and SI_max_ are the minimum and maximum SI values identified on the Pareto front, and CI_min_ and CImax are the minimum and maximum CI values identified on the Pareto front.

## Future Research

This section presents various ideas to further research, which combined could present a future research plan in this important area of research and eventually for adoption by the industry. First, the model would benefit from expanding its search space to include alternative technology choices for the provision of sanitary services. While this is not a typical decision to be made during construction, it would represent an integrated approach to optimizing the slums intervention strategies that span both the design and construction phases and offer a more comprehensive perspective that can eventually identify a low-cost technology solution.

From a modeling and technical point of view, future research will focus on addressing some of the model limitations as well as expanding the model capabilities, including investigating other work propagation techniques in addition to the breadth-first search. Furthermore, BIM can be used to visualize the developed planning and construction scenarios in order to facilitate communication and decision-making among stakeholders. In addition, the decision variables definition can be expanded to consider alternatives to equipment characteristics and construction means. The objective functions definition could be expanded to compute the opportunity cost of the upgrading project. This dimension could look at quantifying the disruptions to slum dwellers due to various construction methods. The model formulation could also be expanded to quantify the risk exposure of proposed construction plans.

In order to showcase the capabilities of the model, case study research could be employed to implement the model on a new real-life project. The objective of this case study would be to observe the document the benefits of apply the model in guiding planning decisions, its practical limitations, and additional cost that could be required in adopting this new model. In such case, the optimized plans generated by the model could be compared to traditional plans and a detailed comparison could be presented to demonstrate the added value of the model.

## Conclusion

Sanitary networks upgrading projects in slum areas and unplanned congested areas are high priority projects on the global scale because of their significance to both the environment and human health. These projects face a number of constructability and socioeconomic challenges. This paper presented a framework and optimization model for planning the sanitary upgrading projects in slums areas. While most relevant research studies adopt a qualitative approach when studying slums upgrading projects, this paper presents a quantitative approach to assess the performance of various construction execution plans. The proposed framework and associated models guide construction planners in selecting construction methods taking into account the road network and equipment characteristics. Furthermore, the research presents an optimization methodology for selecting the optimal sequencing for these challenging upgrading projects that is capable of (1) accelerating the provision of sanitary services to residents and (2) minimizing the net present value of construction costs. To this end, the proposed methodology employs multi-objective genetic algorithms.

The proposed framework and optimization model can serve the various stakeholders engaged in upgrading projects. The contractors can utilize the optimization model to identify the best construction means and methods during early planning phases. The optimization model can be used by local authorities and public agencies to plan the desired development projects and prioritize allocated funds for projects.

A case study is presented to demonstrate the model capabilities using a recent upgrading project in a densely populated slum area. In the presented case study, the model could eliminate more than 3.6 million impractical solutions from the search space. Out of the remaining 864 possible solutions, the model identified 42 Pareto optimal solutions. Based on this specific case study, the spread of the optimal solutions along the cost dimension was insignificant (only 0.3%) compared to the spread along the serviceability dimension (about 3%).

The key contribution of this research to the body of knowledge is in developing a comprehensive construction planning framework capable of achieving the following key objectives. First, the framework accounts for the unique constructability challenges facing sanitary upgrading projects in slums; including logistical challenges and residents’ resistance. Second, the framework is capable of assessing and accelerating the rate of sanitation service provision. Third, the framework is operationalized using a multi-objective optimization model capable of optimizing construction plans.

## References

[1] Brusselaers N (2020). Economic, social and environmental impact assessment for off-site construction logistics: the data availability issue. IOP Conf Ser: Earth Environ Sci..

[2] Maas G, van Gassel F (2005). The influence of automation and robotics on the performance construction. Autom Constr..

[3] Solanki, T.S., R.R.J.I.J.o.S. Salgude, and T. Research, Typical challenges faced in the slum transformation construction project. 2019. 8: 1586-1589.

[4] Akpabio EM (2021). Slums, women and sanitary living in South-South Nigeria. J Hous Built Environ..

[5] Friesen, J.; Knoche, C.; Hartig, J.; Pelz, P.F.; Taubenbock, H.; Wurm, M. Sensitivity of slum size distributions as a function of spatial parameters for slum classification. In *Proceedings of the 2019 joint urban remote sensing event (JURSE)*, Vannes, France, 2019; pp. 1–4.

[6] Un-Habitat (2012). State of the World's Cities 2008/9: Harmonious Cities.

[7] Afshar MH Application of a genetic algorithm to storm sewer network optimization. Sci Iran 2009;13(3):234–44

[8] Pan TC, Kao JJ. GA-QP *model to optimize sewer system design*. J Environ Eng 2009;135(1):17–24

[9] Haghighi A, Bakhshipour AE (2012). Optimization of sewer networks using an adaptive genetic algorithm. Water Resour Manag..

[10] Moeini R, Afshar MH (2013) Sewer network design optimization problem using ant colony optimization algorithm and tree growing algorithm. EVOLVE-A bridge between probability, set oriented numerics, and evolutionary computation IV. Springer, New York, pp 91–105

[11] Safavi H, Geranmehr MA (2017). Optimization of sewer networks using the mixed-integer linear programming. Urban Water J..

[12] Hsie M, Wu M-Y, Huang CY (2019). Optimal urban sewer layout design using Steiner tree problems. Eng Optim..

[13] Zaheri MM, Ghanbari R, Afshar MH (2020). A two-phase simulation–optimization cellular automata method for sewer network design optimization. Eng Optim..

[14] Duque N (2020). Sewer network layout selection and hydraulic design using a mathematical optimization framework. Water.

[15] Abraham DM (1998). Optimization modeling for sewer network management. J Constr Eng Mana..

[16] Halfawy MR, Dridi L, Baker S (2008). Integrated decision support system for optimal renewal planning of sewer networks. J Comput Civ Eng..

[17] Ermolin YA (1999). Mathematical modelling for optimized control of Moscow's sewer network. Appl Math Model..

[18] Ferrada X, Serpell A (2014). Selection of construction methods for construction projects: a knowledge problem. J Constr Eng Manag..

[19] Roy D, Lees M (2020). Understanding resilience in slums using an agent-based model. Comput Environ Urban Syst..

[20] Anwar OE, Aziz TA. An automated collaborative framework to develop scenarios for slums: upgrading projects according to implementation phases and construction planning*.* In *Proceedings of Congress on Computing in Civil Engineering*, 2011;June 19–22, Miami, Florida, United States.

[21] Ibrahim A, El-Anwar O, Marzouk M (2018). Socioeconomic impact assessment of highly dense-urban construction projects. Autom Constr..

[22] El-Anwar O, Aziz TA (2014). Integrated Urban-Construction Planning Framework for Slum Upgrading Projects. J Constr Eng Mana..

[23] Celentano G (2020). The informal city as a socio-technical system: Construction management and money distribution in the informal and upgraded communities of Bangkok. J Clean Prod..

[24] Bahi M, El-Anwar O, Marzouk M. Identifying the optimal execution plan for slum infrastructure upgrading projects. In *Proceedings of construction research congress 2016*. 2016, San Juan, Puerto Rico p. 2070–78.

[25] Katukiza AY (2010). Selection of sustainable sanitation technologies for urban slums — a case of Bwaise III in Kampala Uganda. Sci Total Environ..

[26] Katukiza AY (2012). Sustainable sanitation technology options for urban slums. Biotechnol Adv..

[27] Schouten MAC, Mathenge RW (2010). Communal sanitation alternatives for slums: a case study of Kibera, Kenya. Phys Chem Earth, Parts A/B/C..

[28] Hasan M, Lu M (2018). Planning work for a self-tracking excavator capable of automatic field survey. 17th International Conference on Computing in Civil and Building Engineering.

[29] Han LD, Wang H, Mackey WF (2006). Finding shortest paths under time–bandwidth constraints by using elliptical minimal search area. Transp Res Rec..

[30] Zengin HA, Işik AH. *Improvement for traditional genetic algorithm to use in optimized path finding. In Artificial Intelligence and Applied Mathematics in Engineering Problems*. Springer, Cham, Switzerland; 2020

[31] Deb, K., Multi-objective optimisation using evolutionary algorithms: an introduction, in multi-objective evolutionary optimisation for product design and manufacturing, L. Wang, A.H.C. Ng, and K. Deb, Editors. 2011, Springer London: UK. 3–34.

